# Inhibition of glutamine metabolism counteracts pancreatic cancer stem cell features and sensitizes cells to radiotherapy

**DOI:** 10.18632/oncotarget.5150

**Published:** 2015-09-03

**Authors:** Doudou Li, Zhiqiang Fu, Ruiwan Chen, Xiaohui Zhao, Yu Zhou, Bing Zeng, Min Yu, Quanbo Zhou, Qing Lin, Wenchao Gao, Huilin Ye, Jiajia Zhou, Zhihua Li, Yimin Liu, Rufu Chen

**Affiliations:** ^1^ Department of Radiotherapy, Sun Yat-sen Memorial Hospital, Sun Yat-sen University, Guangzhou, China; ^2^ Department of Hepato-Pancreato-Billiary Surgery, Sun Yat-sen Memorial Hospital, Sun Yat-sen University, Guangzhou, China; ^3^ Department of Radiotherapy, The First Affiliated Hospital, Sun Yat-sen University, Guangzhou, China; ^4^ Department of Medical Oncology, Sun Yat-sen Memorial Hospital, Sun Yat-sen University, Guangzhou, China; ^5^ Department of General Surgery, Guangdong General Hospital, Guangzhou, China; ^6^ Department of Pediatric Surgery, Sun Yat-sen Memorial Hospital, Sun Yat-sen University, Guangzhou, China; ^7^ Key Laboratory of Malignant Tumor Gene Regulation and Target Therapy of Guangdong Higher Education Institutes, Sun Yat-sen Memorial Hospital, Sun Yat-sen University, Guangzhou, China

**Keywords:** pancreatic ductal adenocarcinoma, glutamine metabolism, cancer stem cells, reactive oxygen species, radiosensitivity

## Abstract

Pancreatic ductal adenocarcinoma (PDAC) cells utilize a novel non-canonical pathway of glutamine metabolism that is essential for tumor growth and redox balance. Inhibition of this metabolic pathway in PDAC can potentially synergize with therapies that increase intracellular reactive oxygen species (ROS) such as radiation. Here, we evaluated the dependence of pancreatic cancer stem cells (PCSCs) on this non-canonical glutamine metabolism pathway and researched whether inhibiting this pathway can enhance radiosensitivity of PCSCs. We showed that glutamine deprivation significantly inhibited self-renewal, decreased expression of stemness-related genes, increased intracellular ROS, and induced apoptosis in PCSCs. These effects were countered by oxaloacetate, but not α-ketoglutarate. Knockdown of glutamic-oxaloacetic transaminase dramatically impaired PCSCs properties, while glutamate dehydrogenase knockdown had a limited effect, suggesting a dependence of PCSCs on non-canonical glutamine metabolism. Additionally, glutamine deprivation significantly increased radiation-induced ROS and sensitized PCSCs to fractionated radiation. Moreover, transaminase inhibitors effectively enhanced ROS generation, promoted radiation sensitivity, and attenuated tumor growth in nude mice following radiation exposure. Our findings reveal that inhibiting the non-canonical pathway of glutamine metabolism enhances the PCSC radiosensitivity and may be an effective adjunct in cancer radiotherapy.

## INTRODUCTION

Pancreatic ductal adenocarcinoma (PDAC) is a lethal malignance with a 5-year survival rate under 5% [1]. Approximately 40% of PDAC patients harbor a locally advanced, unresectable, non-metastatic disease termed ‘locally advanced pancreatic cancer’ [2]. Chemoradiation therapy is the conventional option for these patients. However, because of the inherent ability of PDAC to become chemoresistant and radioresistant, such combined modality therapy consistently fails to improve outcomes [3, 4]. Cancer stem cells (CSCs) are defined as a subpopulation within a tumor that contribute to cancer initiation and are highly tumorigenic, can self-renew, and can also develop into differentiated progeny [5]. Emerging evidence indicates that tumor recurrence or metastasis after anticancer treatment is attributable to CSCs [6, 7], and that chemoradiation resistance in PDAC cells may be linked to pancreatic CSCs (PCSCs) [8, 9]. Therefore, novel therapies that overcome PCSC chemoradiation resistance could improve the long-term results of PDAC therapy.

Glutamine metabolism is recognized as a central metabolic pathway in tumors, contributing to oncogenic transformation and maintenance and fueling tumor growth [10]. PDAC cells have been recently found to utilize a non-canonical glutamine metabolism pathway that is essential for PDAC tumor growth [11]. This pathway appears dispensable in normal cells and therefore might provide an accessible therapeutic entry point to treat PDAC [12]. Importantly, this non-canonical pathway is essential for maintaining redox balance and preventing excessive generation of intracellular reactive oxygen species (ROS) [11, 13]. Since radiotherapy relies on ROS toxicity to eradicate tumor cells [14, 15], blocking this non-canonical glutamine metabolism pathway may efficiently increase the radiosensitivity of PCSCs.

In this study, we first clarified that inhibition of the non-canonical glutamine metabolism pathway impaired the properties of PCSCs, increased the generation of intracellular ROS, and dramatically promoted the anti-tumor effect of IR treatment in PCSCs both *in vitro* and *in vivo*. Moreover, glutaminase inhibitors, including Zaprinast and BPTES, could effectively sensitize PCSCs to IR and increase apoptosis via intracellular ROS accumulation. Our findings suggest that blocking glutamine metabolism represents a promising therapeutic strategy to sensitize pancreatic cancer cells to IR treatment by disrupting redox balance.

## RESULTS

### PCSC growth is dependent on glutamine

**Figure 1:**
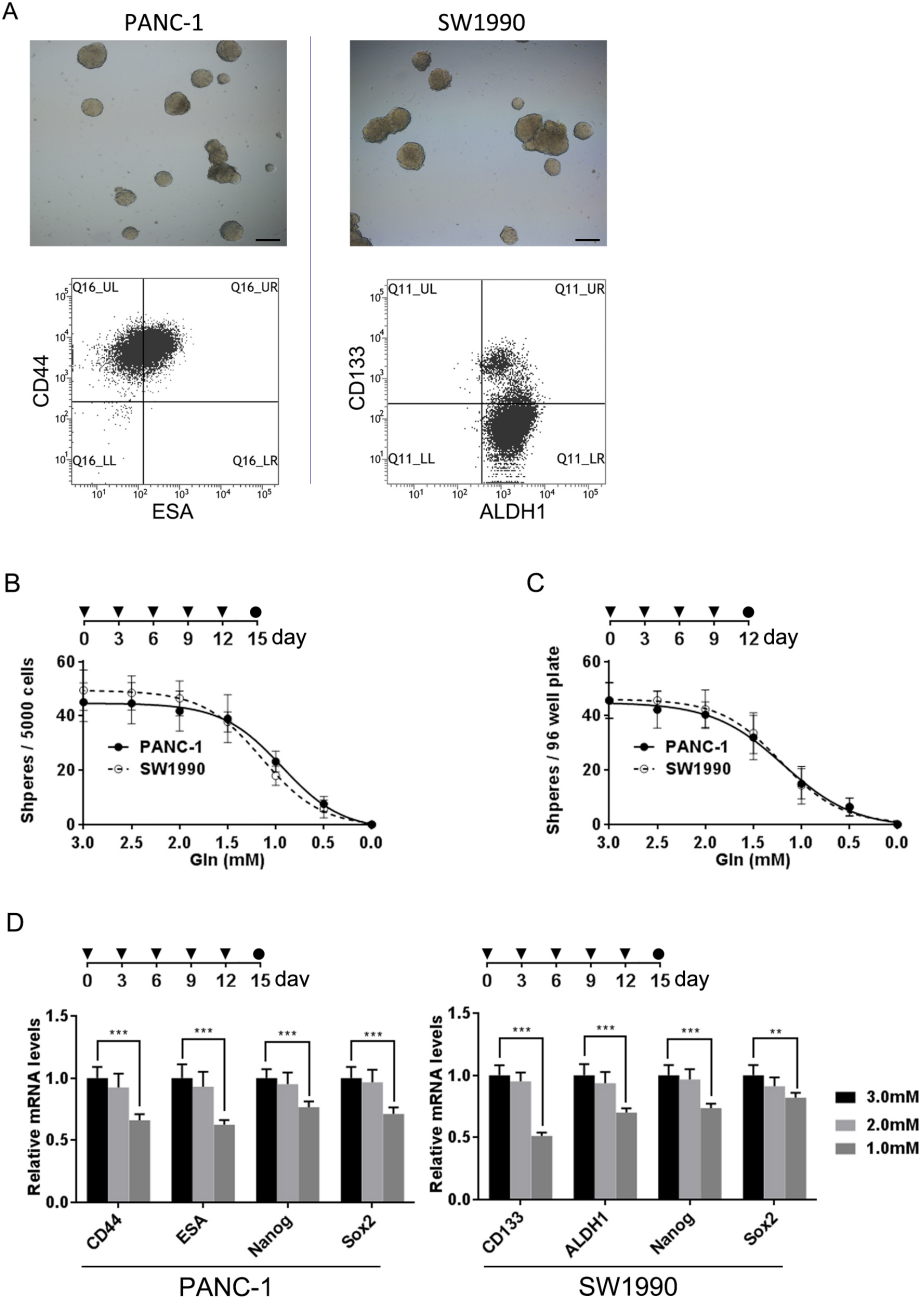
PCSCs are dependent on glutamine **A.** representative images of spheres derived from PANC-1 and SW1990 cells; subpopulations of PCSCs were evaluated via flow cytometry analysis. Scale bar: 100 μm. **B.** sphere formation assays were performed in the presence of different glutamine concentrations and sphere formation capacity was quantified by calculating the number of spheres per 5000 single cells. **C.** the effect of glutamine deprivation on secondary sphere formation was quantified by calculating the number of spheres formed from 100 single cells of passage one spheres. **D.** the effect of glutamine deprivation on the expression of indicated PCSC-related genes in spheres was measured by qRT-PCR. Error bars represent the mean ± S.D. of triplicate experiments. Statistical significance was calculated using the Student's *t* test or ANOVA. **p < 0.01; ***p < 0.001. ▼ replacing culture medium; ●, timing of evaluation.

Tumor cell dependence on exogenous glutamine varies among different tumors [16]. Recent evidence indicates that glutamine metabolism is required for pancreatic cancer growth [11]. However, whether PCSCs are dependent on glutamine (Gln) metabolism is unknown. To assess the dependence of PCSCs on glutamine, we first conducted sphere-formation culture using PANC-1 and SW1990 cells, as the sphere-forming process should enrich potential CSC subpopulations [17, 18]. Spheres were obtained after 2 weeks of culture (Fig. 1A), and we evaluated mRNA expression of the CSC markers CD44, ESA, CD133, ALDH1, Nanog, SOX2, and Nestin in cells within the spheres. Sphere-forming cells had enriched expression of all CSC markers (Supplementary Fig. 1A). Notably, CD44 and ESA were particularly overexpressed in PANC-1 spheres, while SW1990 spheres showed high expression levels of ALDH1 and CD133. Consistently, flow cytometry assays demonstrated a dominant CD44+/ESA+ subpopulation in PANC-1 spheres, and a large ALDH1+/CD133+ subpopulation in SW1990 cells (Fig. 1A). The high tumorigenic capacity of these cells—another property of cancer stem cells—was confirmed by nude mouse tumorigenicity assay (Supplementary Fig. 1B).

The importance of glutamine metabolism in PCSCs was evaluated by first exploring the effect of glutamine deprivation on sphere formation. The total number of primary spheres formed after 2 weeks of culture was reduced when the concentration of glutamine was decreased (Fig. 1B). Meanwhile, primary spheres formed in medium with a normal glutamine concentration (2.5 mM) were subjected to a secondary sphere-formation assay with different glutamine concentrations. Consistently, a decreased glutamine concentration also impaired secondary sphere formation capacity (Fig. 1C). Additionally, glutamine deprivation reduced levels of PCSC marker expression in spheres (Fig. 1D). Taken together, these data indicate that glutamine is necessary for sphere formation, maintenance of PCSC marker expression, and the self-renewal capacity of PCSC.

### The non-canonical glutamine metabolism pathway is critical for PCSCs

**Figure 2:**
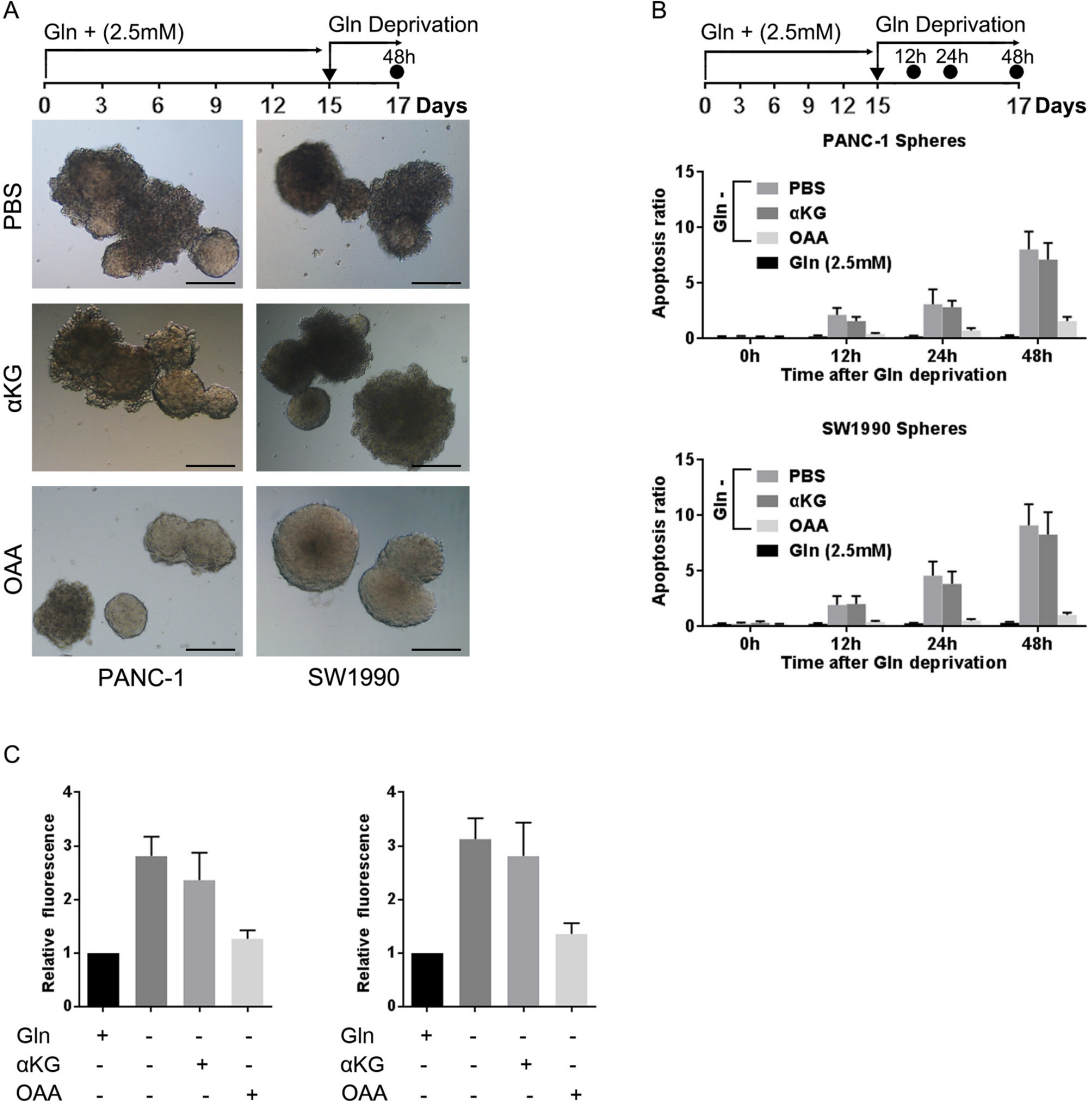
OAA restores the adverse effects of glutamine deprivation on spheres **A.** representative images showing the effect of supplementation with PBS, dimethyl αKG, or OAA in glutamine-free medium on the morphological features of spheres. Scale bar: 100 μm. **B.** apoptosis of sphere cells after 48 h culture in glutamine-free medium supplemented with the indicated substrates was evaluated by flow cytometry. **C.** ROS production as evaluated by measuring DCF fluorescence via fluorescence microscopy after 24 h glutamine deprivation and subsequent supplementation with glutamate, dimethyl αKG, or OAA. The ROS levels of spheres cultured in medium with normal glutamine supplement were set as 1. Error bars represent the mean ± S.D. of triplicate experiments. Statistical significance was calculated using the Student's *t* test or ANOVA. **p < 0.01; ***p < 0.001. ▼, replacing culture medium supplemented with the indicated nutrients; ●, timing of evaluation.

PDAC cells utilize glutamine (Gln) primarily by the non-canonical pathway, different from other glutamine-dependent tumors in which GLUD1 predominates. However, whether PCSCs utilize glutamine in the same manner as PDAC cells has not been addressed. We cultured spheres derived from PANC-1 and SW1990 cells in glutamine-free medium supplemented with either dimethyl αKG or oxaloacetate (OAA), which are products of canonical and non-canonical glutamine metabolism, respectively. The morphology of spheres was obviously changed after 48 h of glutamine deprivation, and dimethyl αKG did not prevent spheres from dissociating (Fig. 2A). In contrast, supplementation with OAA maintained the features of spheres following glutamine deprivation (Fig. 2A). Additionally, we observed a mild increase in apoptosis following by glutamine deprivation, with OAA—but not dimethyl αKG—preventing apoptosis following glutamine deprivation (Fig. 2B). Given the importance of glutamine metabolism in maintaining the redox state of PDAC, we next examined the effect of glutamine deprivation on ROS levels in PCSCs. glutamine deprivation significantly increased the ROS level, and OAA—but not dimethyl αKG—was capable of restoring ROS to normal levels (Fig. 2C).

**Figure 3:**
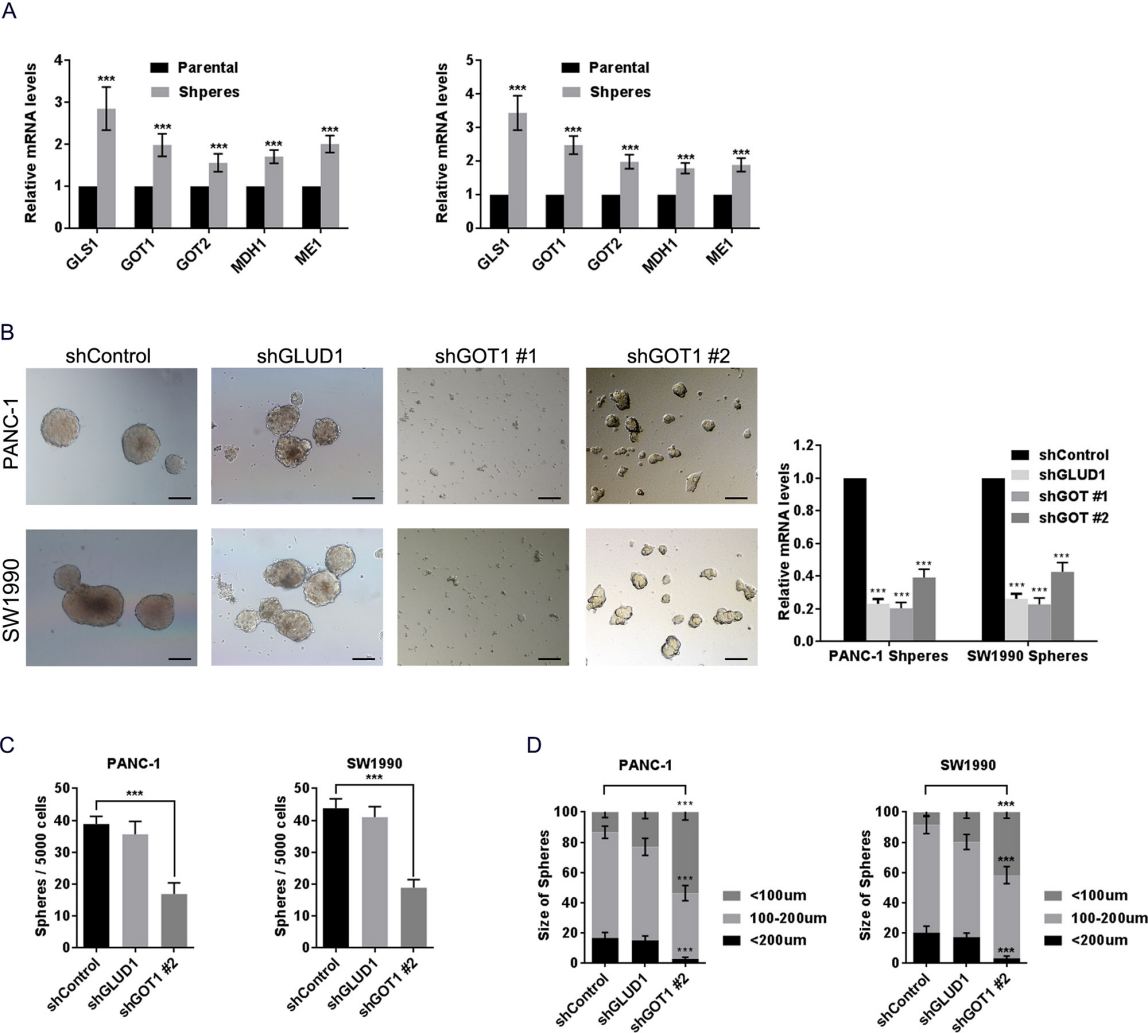
Inhibition of non-canonical glutamine metabolism impaired sphere formation **A.** mRNA levels of GLUD1, GLS1, GOT1, GOT2, MDH1, and ME1 in spheres compared with levels in parental adherent cells as measured by qRT-PCR. **B.** PANC-1 and SW1990 cells were transfected with control shRNA or shRNA against either GLUD1 or GOT1. Images show the representative sphere features in each group following culture in sphere formation medium for 14 days. Knockdown efficiencies of shRNAs are shown in the right panel. Scale bar: 100 μm. **C.** number of spheres formed following GLUD1- and GLS1-knockdown in PANC-1 and SW1990 cells. **D.** size of spheres formed following GLUD1- and GLS1-knockdown in PANC-1 and SW1990 cells. Error bars represent the mean ± S.D. of triplicate experiments. Statistical significance was calculated using the Student's *t* test or ANOVA. **p < 0.01; ***p < 0.001.

We further examined the importance of the non-canonical glutamine pathway in PCSCs by measuring expression of key enzymes involved in this pathway at the mRNA level. Sphere-forming cells had elevated expression levels of all enzymes assessed (Fig. 3A). Next, expression of GLUD1 or glutamic-oxaloacetic transaminase (GOT1; responsible for production of OAA from glutamine) expression in PCSCs was suppressed by lentivirus-mediated shRNAs transfection prior to sphere-formation assays (Supplementary figure 2). GLUD1 suppression had very mild influence on sphere formation in terms of morphology (Fig. 3B), total number (Fig. 3C), and size (Fig. 3D). In contrast, knockdown of GOT1 expression to approximately 20% the level of control (using shGOT1 #1) dramatically impaired sphere formation (Fig. 3B). These cells treated with shGOT1#1 tended to remain as single cells without forming spheres. We also used a relatively low efficiency shRNA (shGOT1#2) to suppress GOT1, and found that a 50–60% suppression of GOT1 also obviously impaired sphere formation and induced abnormal morphological features in the spheres that formed (Fig. 3B). Consistently, a reduction in the number and size of spheres was observed following GOT1 knockdown with shGOT1#2 (Fig. 3C, 3D).

### Inhibiting the non-canonical glutamine metabolism pathway sensitizes PCSCs to radiation via intracellular ROS accumulation

**Figure 4:**
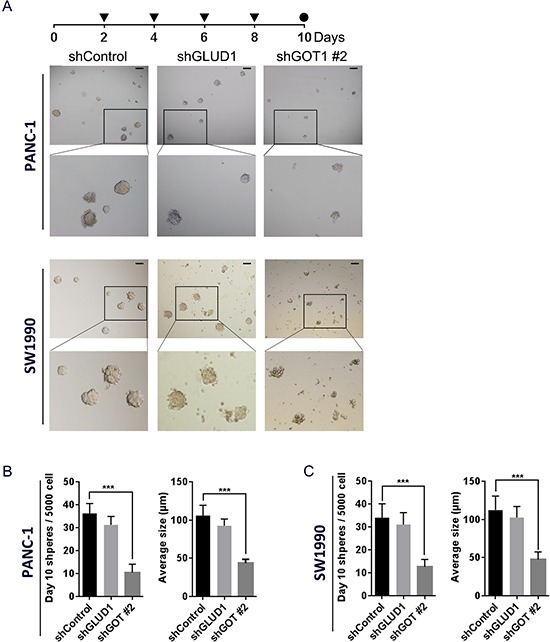
Inhibition of non-canonical glutamine metabolism impairs sphere formation following continuous lose-dose IR treatment **A.** bright-field microscopy images showing features of shControl, shGLUD1, and shGLS1#2 pancreatic cancer cell-derived spheres following sphere-formation culture with 2 Gy IR treatment every second day. Scale bar: 200 μm. **B.** quantification of total number and average size of PANC-1 spheres in 4A after 10 days of sphere-formation culture. **C.** quantification of total number and average size of SW1990 spheres in 3A after 10 days of sphere-formation culture. Error bars represent the mean ± S.D. of triplicate experiments. Statistical significance was calculated using the Student's *t* test or ANOVA. **p < 0.01; ***p < 0.001.

We have previously shown that inhibiting the non-canonical pathway of glutamine metabolism increases ROS levels in PCSCs. Consistently, blockage of the canonical pathway had only a minimal impact on ROS levels. Therefore, we speculated that the non-canonical pathway of glutamine metabolism may be associated with cellular radiosensitivity. To address this, we first evaluated the effect of low-dose IR (2 Gy) on sphere formation in shControl, shGLUD1, and shGOT1#2 PCSCs. We observed a modest reduction in both size and number of spheres following GLUD1 knockdown and 2 Gy IR every 2 days (Fig. 4A–4C). In contrast, GOT1 knockdown in combination with 2 Gy IR every second day significantly impaired sphere formation, and cells in these spheres underwent apoptosis approximately ten days following sphere formation (Fig. 4A–4C). These results suggest that inhibition of non-canonical glutamine metabolism cooperates with low-dose IR to impair sphere formation in a synergistic manner.

**Figure 5:**
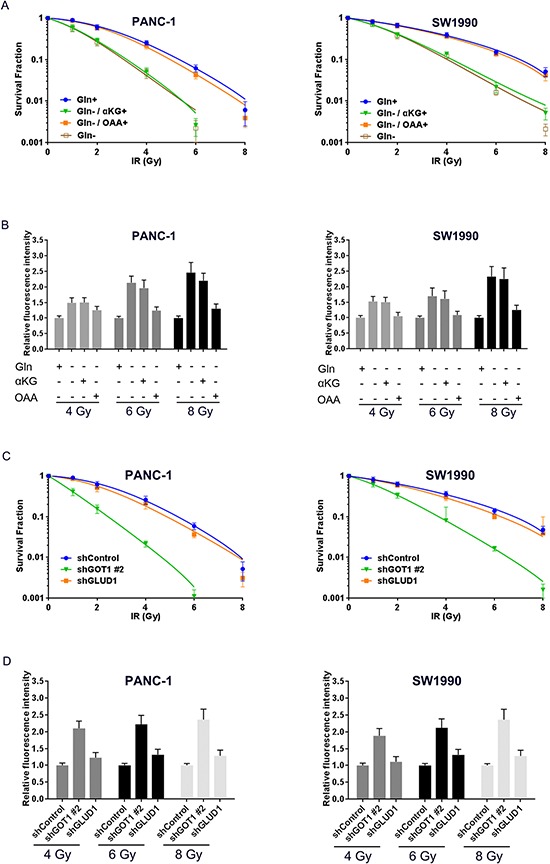
Inhibition of non-canonical glutamine metabolism sensitizes PCSCs to radiotherapy via intracellular ROS accumulation **A.** PANC-1 (left panel) and SW1990 (right panel) spheres were obtained by 14 days of culture, and the culture medium was replaced with fresh medium containing indicated supplements (glutamine, 2.5 mM; dimethyl αKG, 4 mM; OAA, 4 mM) 12 hours before IR treatment. Clone formation was assessed 14 days after irradiation and survival fractions were calculated for each group. **B.** sphere cells received treatment as described in 5A and the relative DCF fluorescence measured after exposure to 4, 6, or 8 Gy IR. Fluorescence intensity of cells cultured in medium supplemented with 2.5 mM glutamine for each IR group was set as 1. **C.** shControl, shGLUD1, and shGLS1#2 cells were cultured in sphere-formation condition for two weeks followed by treatment with indicated IR doses. Clone formation was measured 14 days after irradiation. Survival fractions were calculated for each group. **D.** cells treated as described in 5C were treated with 4, 6, or 8 Gy IR followed by measurement of DCF fluorescence. The fluorescence intensity of shControl group was set as 1. Error bars represent the mean ± S.D. of triplicate experiments. Statistical significance was calculated using the Student's *t* test or ANOVA. **p < 0.01; ***p < 0.001. ▼, irradiation treatment; ●, timing of evaluation.

We next used clonogenic survival assays to confirm the synergistic effect of blocking non-canonical glutamine metabolism on IR sensitization. We irradiated cells incubated in medium supplemented with or without glutamine, and found that glutamine deprivation significantly promoted radiosensitivity. Meanwhile, glutamine deprivation-induced radiosensitivity was abrogated by OAA (Fig. 5A). Furthermore, glutamine deprivation significantly increased the intracellular ROS levels upon IR treatment, and OAA clearly prevented ROS generation (Fig. 5B). Additionally, PCSCs carrying shControl, shGLUD1, and shGOT1#2 were subjected to 2 weeks of culture with fractional IR treatment, and suppression of GOT1—but not GLUD1—significantly increased radiosensitivity in PCSCs from both pancreatic cancer cell lines (Fig. 5C). Meanwhile, GOT1 suppression significantly increased intracellular ROS levels (Fig. 5D). Taken together these findings indicate that blocking the non-canonical pathway of glutamine metabolism can promote radiosensitivity in PCSCs by increasing ROS generation.

### Glutaminase inhibition promotes radiosensitivity of PCSCs *in vitro* and *in vivo*

**Figure 6:**
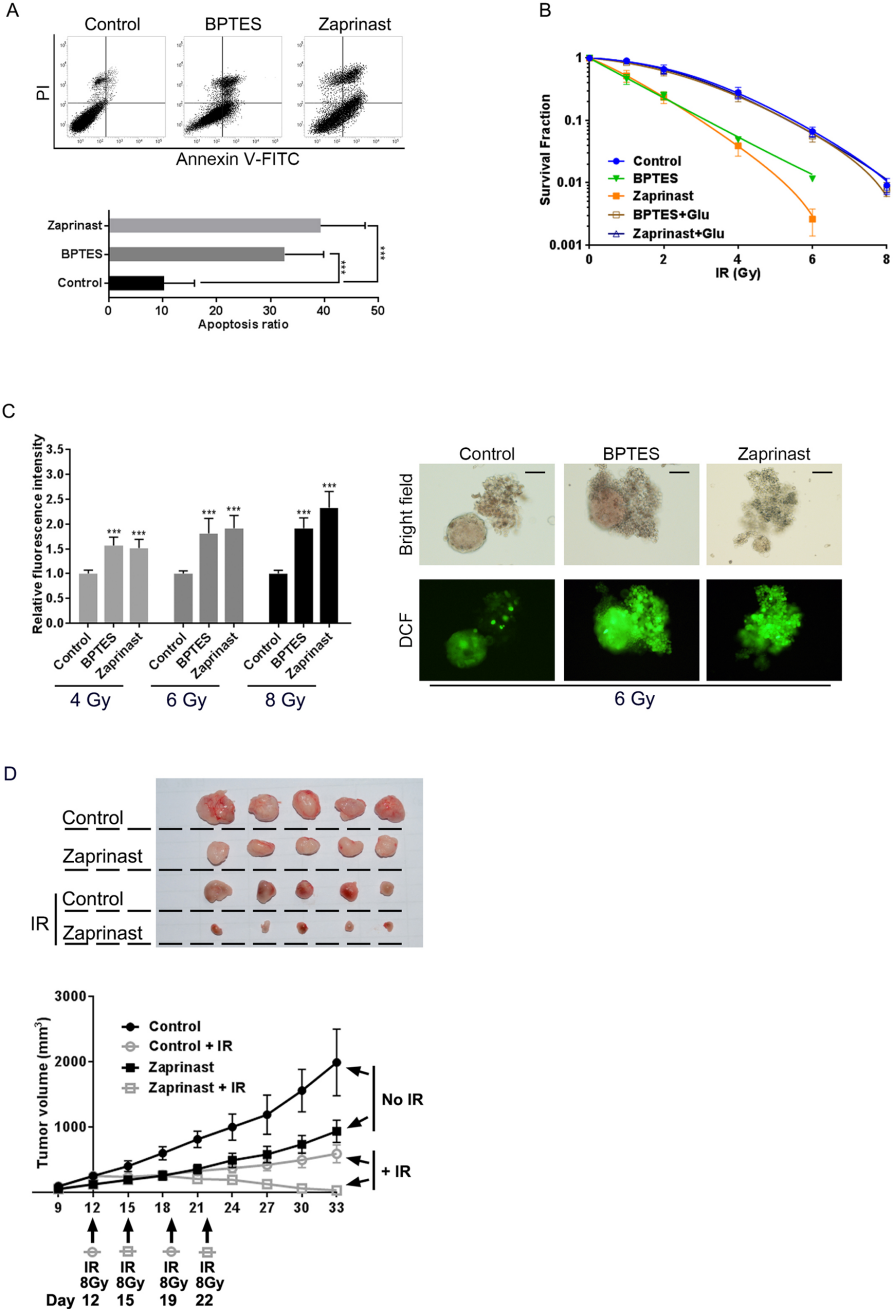
Glutaminase inhibition enhanced radiosensitivity of PCSCs *in vitro* and *in vivo* **A.** PANC-1 spheres were treated with BPTES (200 nM) or Zaprinast (500 nM) for 12 h before exposure to 6 Gy IR. Apoptosis was evaluated by flow cytometry analysis 6 h after IR treatment. **B.** PANC-1 spheres were treated with BPTES (200 nM) or Zaprinast (500 nM) for 12 hours before IR treatment, and clone formation was assessed 14 days after irradiation. Survival fractions were calculated for each group. **C.** cells received IR treatment as described in 6B and relative DCF fluorescence was measured. Representative bright field micrographs show typical morphology changes, and fluorescence images reflect intracellular ROS accumulation, after 6 Gy IR treatment. Scale bar: 100 μm. **D.** nude mice were subcutaneously injected (in the right axilla) with 1 × 10^5^ cells from PANC-1 spheres. Peritumor areas were then injected with 20 μl Zaprinast (600 μM) or PBS by every 2 days. Tumors were then exposed to an 8-Gy dose of radiation twice a week. Tumor volume was monitored using calipers by once every three days from days 9–33. Data are presented as tumor growth curves. Irradiation treatment times are indicated by arrows labeled with legends indicating the treated group.

Several glutaminase inhibitors have been developed, including 968 and BPTES [19–21]. Additionally, the asthma medication Zaprinast has been recently identified as a glutaminase inhibitor [22]. Therefore, we hypothesized that disruption of glutamine metabolism with glutaminase inhibitors could abrogate the growth of pancreatic CSCs. Compared with control, both BPTES and Zaprinast significantly promoted apoptosis induced by 6 Gy IR (Fig. 6A). Additionally, BPTES and Zaprinast increased radiosensitivity in PCSCs (Fig. 6B). Generation of IR-induced intracellular ROS was also enhanced upon BPTES and Zaprinast treatment (Fig. 6C). We selected Zaprinast for *in vivo* experiments on account of its superior physicochemical properties, in particular solubility. Consistent with *in vitro* findings, Zaprinast increased the sensitivity of PANC-1 sphere-derived tumors to IR treatment *in vivo* (Fig. 6D). This suggests that combined treatment with Zaprinast and IR could inhibit tumor growth effectively.

## DISCUSSION

While they represent only a miniscule proportion of the total cancer cell population, CSCs are believed to be the source of tumor recurrence [23]. Efforts to develop novel therapeutic strategies to improve the poor prognosis of PDAC have focused on targeting PCSCs. Here, we have investigated the utility of a dual-modality therapy that targets PCSCs. We have uncovered a relationship between glutamine metabolism and resistance to IR in PCSCs. We found that the non-canonical glutamine metabolism pathway is critical for maintaining the properties of PCSCs. Furthermore, we confirmed that blockage of this glutamine metabolism pathway sensitizes pancreatic cancer cells to IR by enhancing ROS generation. Finally, we found that the glutaminase inhibitors BPTES and Zaprinast could promote radiosensitivity of PCSCs. Therefore, we have identified synergy between blocking glutamine metabolism and radiotherapy for treatment targeting PCSCs. This finding may have significant therapeutic implications, especially given that clinical grade glutaminase inhibitors are being developed [24].

The importance of glutamine as a nutrient in cancer derives from its ability to supply nitrogen and carbon atoms to an array of growth-promoting pathways [25]. Glutamine can be converted into αKG to replenish TCA cycle metabolites through either GLUD1 or transaminase action [26]. Most cancers depend on GLUD1-mediated glutamine metabolism to fuel the TCA cycle [27]. However, PDAC cells metabolize glutamine in a manner that relies on transaminases, not GLUD1 [11]. In this non-canonical pathway, GOT1 converts glutamine-derived aspartate to OAA, which can be used to generate malate and pyruvate. This chain of reactions occurs with assistance from other transaminases, including GLS1, GOT2, MDH1, and ME1 to increase NADPH levels and help maintain the intracellular redox balance [28]. We observed increased expression of transaminases in PCSCs compared with parental cell lines, suggesting that this non non-canonical pathway is necessary for maintaining of the cancer stem cell properties in PCSCs. We also found that PCSC properties depend on non-canonical glutamine metabolism, suggesting glutamine metabolism is a worthy therapeutic target in PCSCs.

Glutamine metabolism was also required for limiting ROS generation in PCSCs as is the case for cultured pancreatic cancer cells [11]. ROS regulate a broad array of signal transduction pathways in multiple biological processes, including cell growth, differentiation, gene expression, and apoptosis. ROS production contributes to tumor cell apoptosis following exposure to IR and other stressors such as high glucose, angiotensin, and tumor necrosis factor α [29]. CSCs are more radioresistant than non-CSCs, and this is partly attributable to the lower ROS levels and enhanced ROS defenses found in CSCs [30]. However, Son et al. found that PDAC cells are markedly more sensitive to ROS when glutamine metabolism is impaired [11]. Consistently, we found that glutamine utilization—which is crucial for cell survival upon radiotherapy [31]—could be disrupted to increase ROS generation. glutamine metabolism contributes further to malignancy by sustaining proliferative signaling [32, 33], enabling replicative immortality [34], and resisting cell death [35]. Therefore, blocking the non-canonical glutamine pathway may have significant therapeutic benefit when used in conjunction with radiotherapy or other strategies that generate ROS.

The clinical application of glutamine metabolism as an anti-tumor target is dependent on the successful development of clinical-grade glutamine metabolism inhibitors. Efforts to inhibit glutamine metabolism using amino acid analogs have an extensive history, but all have exhibited variable degrees of toxicity [36]. Therefore, recent work has focused on developing methods directed towards specific nodes of glutamine metabolism [16]. As the non-canonical glutamine metabolism pathway appears dispensable in normal cells, it represents an accessible therapeutic target [11, 12]. Disrupting this pathway requires that inhibitors of the transaminases involved in this non-canonical pathway be developed. Following cellular uptake of glutamine, a significant fraction is converted to glutamate and ammonia by glutaminase (GLS) in the mitochondria [37]. GLS1 expression is elevated in many primary tumors and tumor cell lines, while GLS2 expression appears to be relatively limited in cancer [38]. Two glutaminase inhibitors, 968 and BPTES, have been characterized in recent years [19, 39]. GLS1 could be considered as the upstream glutaminase responsible for the non-canonical utilization of glutamine, and Son et al. successfully inhibited this pathway using either 968 or BPTES *in vitro* [11]. Additionally, an off-target effect of the anti-asthmatic Zaprinast is an increase in ROS levels, which sensitizes cells to oxidative damage by H_2_O_2_ in a manner that can be rescued by extracellular glutamate [22]. We found that Zaprinast had a similar effect on PCSCs. We conducted *in vivo* experiments to examine whether combining a glutamine metabolism inhibitor with IR could yield synergistic anti-tumor effects. Zaprinast dramatically sensitized PCSCs to IR treatment *in vivo*. Our findings support the potential use of glutamine inhibitors with radiotherapy as a therapeutic strategy for PDAC.

Our data confirm that PCSCs are dependent on the non-canonical glutamine metabolism pathway. Moreover, our findings demonstrate the remarkable potential anti-tumor effect of combining a glutamine metabolism inhibitor with radiotherapy in the treatment of PDAC. The development of clinical-grade glutamine inhibitors that target the non-canonical pathway has great potential to improve the clinical efficacy of radiotherapy for PDAC.

## MATERIALS AND METHODS

### Cell culture

The human pancreatic cancer cell lines PANC-1 and SW1990 were obtained from American Type Culture Collection (Manassas, VA, USA) and grown in complete growth medium as recommended by the manufacturer and maintained in a humidified 5% CO_2_ atmosphere at 37°C. To propagate the CSC-like fraction of the tumor cells, culture conditions favoring proliferation of undifferentiated cells were adopted [17, 18]. Briefly, pancreatic cancer cells were cultured in DMEM medium containing 100 U/mL penicillin (Sigma, St. Louis, MO, USA), 100 μg/mL streptomycin (Sigma, St. Louis, MO, USA), bovine serum albumin Fraction V (Sigma), 1× B-27 supplement (Gibco, Carlsbad, CA, USA), 20 ng/mL epidermal growth factor (Invitrogen, Carlsbad, CA, USA), and 20 ng/mL fibroblast growth factor (Invitrogen) in low-attachment dishes (Corning, Corning, NY, USA) at a concentration of 1 × 10^4^ cells/mL.

### Vector construction and virus infection

For lentivirus-mediated suppression of GLUD1, the following GLUD1 shRNA and scrambled control shRNA sequences were inserted into the pMKO.1-puro vector obtained from Invitrogen: GLUD1 shRNA, forward, 5′-CCGGGCAGAGTTCCAAGACAGGATACTCGAG TATCCTGTCTTGGAACTCTGCTTTTTG-3′ and reverse 5′-AATTCAAAAAGCAGAGTTCCAAGACAGGATA CTCGAGTATCCTGTCTTGGAACTCTGC-3′; scrambled control, forward, 5′-CCGGTTTCTCCGAACGTGTCA CGTCTCGAGA CGTGACACGTTCGGAGAATTTT TG-3′ and reverse, 5′-AATTCAAAAAGTTCTCCG AACGTGTCACGTC TCGAGACGTGACACGTTC GGAGAA-3′. For lentivirus-mediated suppression of GOT1, a high knockdown efficiency shRNA and a relatively low efficiency shRNA were inserted into the pMKO.1-puro vector: high efficiency shGOT1#1 forward, 5′-CCGGGCTAATGACAATAGCCTAAATCTCGAG ATTTAGGCTATTGTCATTAGCTTTTTG-3′ and reverse, 5′-AATTCAAAAAGCTAATGACAATAGCCTAAATC TCGAGATTTAGGCTATTGTCATTAGC-3′; relatively low efficiency shGOT1#2 forward, 5′-CCGGGCGTT GGTACAATGGAACACTCGAGTG TTCCATTGTAC CAACGCTTTTTG-3′ and reverse, 5′-AATTCAAAAAGC GTTGGTACAATGGAACACT CGAGTGTTCCATTGT ACCAACGC-3′. Lentivirus packaging, cell infection, and selection of puromycin-resistant cells were performed as previously described [40].

### RNA isolation, microarrays, and quantitative real-time reverse transcription-PCR

Total RNA was extracted from breast tissue specimens or cells using the Trizol reagent (Invitrogen) following manufacturer's instructions. Total RNA was converted to cDNA by reverse-transcription using oligodT primers and SuperScript II reverse transcriptase (Invitrogen). Quantitative real time PCR was carried out on a Roche Light-Cycler system (Roche, Basel, Switzerland) using a SYBR Green reaction mix (Qiagen Sciences, Germany). Relative expression values were calculated using the ΔΔCT method, with GAPDH serving as control. The primer sequences used in the study are listed in Supplementary Table S1.

### Flow cytometry analysis

Detection of CSC markers was performed using CD44-FITC/APC (Becton Dickinson, Auburn, CA, USA), ESA-FITC (StemCell Technologies, Vancouver, BC, Canada), ALDH1-PE (Miltenyi Biotec, Auburn, CA, USA), and CD133/1-PE (Miltenyi Biotec) antibodies. Approximately 1 × 10^6^ cells were distributed into tubes containing PBS with 2% fetal bovine serum and kept on ice for 10 min. Antibodies were added to cell suspensions and incubated on ice in the dark for 30 min. Cells were washed and re-suspended in 500 mL FACS buffer and analyzed using a flow cytometer (BD Biosciences, CA, USA).

### Measurement of intracellular ROS

Intracellular ROS levels were measured using the cell-permeable probe CM-H2DCFDA (Sigma) dissolved in high quality anhydrous dimethylsulfoxide (DMSO). Three replicates were assessed for each observation point. Cells were treated with either CM-H2DCFDA (final concentration 10 μM) or non-oxidizable control (final concentration 2 μM) probe at 1 h after IR and then incubated in 5% CO_2_ at 37°C. Cells were collected after 30 min and washed three times with cold phosphate buffered saline (PBS). Cells were then resuspended in cold PBS at a concentration of 1 × 10^6^ cells/mL, and fluorescence was detected by flow cytometry. For fluorescence imaging, residual medium was removed and replaced with PBS gently, and images were captured using a scanning confocal microscope (LSM710, Carl Zeiss, Thornwood, NY, USA).

### Sphere-formation assays

Single-cell suspensions (1 × 10^4^ cells/mL) were plated on ultralow attachment six-well plates (Corning) and grown in modified DMEM without serum supplementation. Media was replaced every 3 days. Spheres were counted after 14 days (passage one, P1). The number of secondary spheres (passage two, P2) formed following a further 10–12 day incubation after scattering was also counted.

### Irradiation and clonogenic assay

Cells were treated with a single dose of radiation (0, 2, 4, 6, or 8 Gy) and then seeded into 6-well tissue culture plates and incubated for 14 days without changing the culture medium. Cells were then fixed with methanol and stained with crystal violet. The number of colonies with > 50 cells was counted under a dissecting microscope. Cell survival was determined by means of colony formation assay. The plating efficiency (PE) and survival fraction (SF) were calculated as follows: PE = (number of colonies/inoculating cell number) × 100%; SF = number of colonies counted/(cells seeded × (PE/100)).

### *In vivo* tumorigenesis assay

Five-week-old male BALB/c nude mice were obtained from the Laboratory Animal Center, Zhongshan Medical School of Sun Yat-sen University (Guangdong, China) and housed in laminar flow cabinets under specific pathogen-free conditions. All experimental procedures involving animals were in accordance with the Guide for the Care and Use of Laboratory Animals and were performed according to the institutional ethical guidelines for animal experiments. The study protocol was also approved by the Committee on the Use of Live Animals in Teaching and Research, Sun Yat-sen Memorial Hospital, Sun Yat-sen University. PANC-1 cells and PANC-1-derived spheres were collected, enzymatically dissociated, washed in PBS, counted, and injected into the right axilla of each mouse. Tumor size was measured using the following formula: volume = (L × W^2^)/2, where L and W are the longest and shortest diameters of the tumor, respectively. The pharmacokinetics of Zaprinast (Sigma) for use in mice have not been characterized, though we performed direct peritumoral injection of Zaprinast in a volume of 20 μL (600 μM, every 2 days, dissolved in saline supplemented with DMSO) to ensure delivery of the compound based on previous studies and our own pre-experimental testing [22, 41, 42]. Zaprinast injections began when the average tumor volume reached approximately 100 mm^3^. Control mice were injected with an equal volume of saline supplemented with DMSO. Once the average tumor volume reached approximately 300 mm^3^, tumors were irradiated with an 8-Gy dose of radiation sourced from ^60^Co. A second 8-Gy dose was administered a week later. Mice were sacrificed when the largest tumors reached 1.5 cm in diameter. The tumors were then removed and weighed. Each group contained a minimum of five mice.

### Statistical analysis

Statistical evaluation of data was performed using one way ANOVA and Student's *t*-tests where appropriate. The threshold for statistical significance was set at *p* < 0.05

## SUPPLEMENTARY FIGURES AND TABLES


