# Supplementary material for: Inhibition of glutamine metabolism counteracts pancreatic cancer stem cell features and sensitizes cells to radiotherapy
Source: Oncotarget. 2015 Sep 3;6(31):31151–63. doi: 10.18632/oncotarget.5150 (PMC4741594; doi:10.18632/oncotarget.5150)
Supplement: Supplementary file 1 [file oncotarget-06-31151-s001.pdf]

SUPPLEMENTARY FIGURES AND TABLES

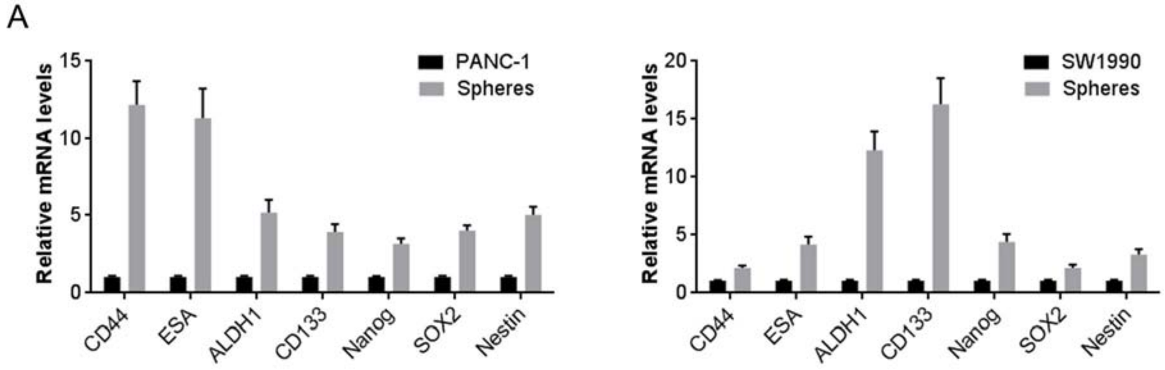

B

Number of cells injected	PANC-1 CSCs	SW1990 CSCs
5×10^4	4/4	4/4
1×10^4	3/4	3/4
5×10^3	04/	1/4

Supplementary Figure S1: A. Relative mRNA expression of stemness-related genes (normalized to GAPDH) in PANC-1 (left panel) and SW1990 cells (right panel) as evaluated by qRT-PCR (spheres vs. parental cell line). B. Indicated numbers of spheroids cells were injected subcutaneously into nude mice, and the tumorigenicity rates are shown.

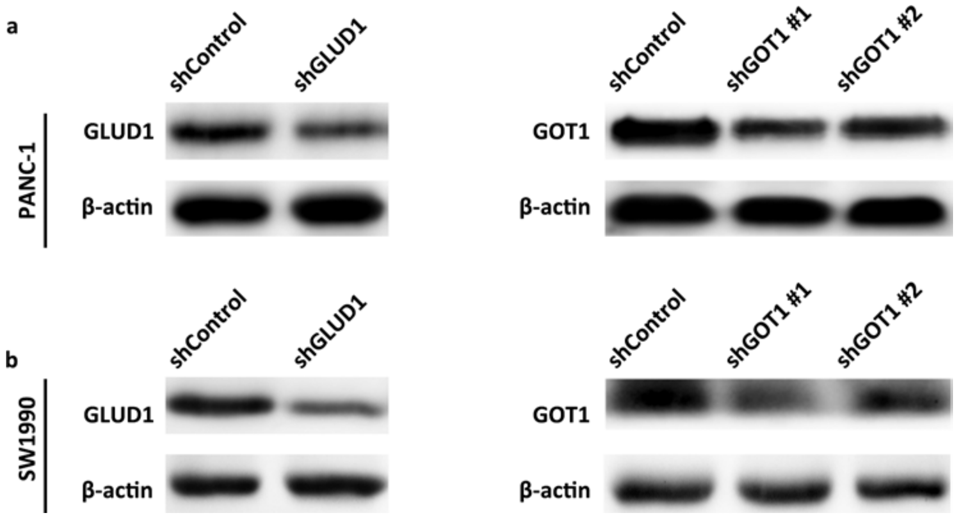

Supplementary Figure S2: A. Knockdown efficiencies of shRNA against GLUD1 and GOT1 in PANC-1 cells. B. Knockdown efficiencies of shRNA against GLUD1 and GOT1 in SW1990 cells.

Supplementary Table S1: The sequences of PCR primers

ALDH1	Forward Primer	5′-	GTT CCT GGT TAT GGG CCT AC	-3′
	Reverse Primer	5′-	CCT GGA TGC GGC TAT ACA AC	-3′
CD133	Forward Primer	5′-	GGTCTGGCGAGCTAAGGGAA	-3′
	Reverse Primer	5′-	GGGGAAGGCAAGCGTGTT	-3′
CD44	Forward Primer	5′-	TTTGCATTGCAGTCAACAGTC	-3′
	Reverse Primer	5′-	GTTACACCCCAATCTTCATGTCCAC	-3′
ESA	Forward Primer	5′-	ACCTGCTCTGAGCGAGTGAGAACCT	-3′
	Reverse Primer	5′-	GACCAGGATCCAGATCCAGTTG	-3′
GLS1	Forward Primer	5′-	GCTTTCCATGTTGGTCTTCC	-3′
	Reverse Primer	5′-	AAACAAGATCGTGACAAAAGTGAA	-3′
GLUD1	Forward Primer	5′-	GGGATTCTAACTACCACTTGCTCA	-3′
	Reverse Primer	5′-	AACTCTGCCGTGGGTACAAT	-3′
GOT1	Forward Primer	5′-	CAACTGGGATTGACCCAACT	-3′
	Reverse Primer	5′-	GGAACAGAAACCGGTGCTT	-3′
GOT2	Forward Primer	5′-	GGATCTGGAGGTCCCATTTC	-3′
	Reverse Primer	5′-	ATGGCCCTGCTGCACTC	-3′
MDH1	Forward Primer	5′-	TAAGGTTATCGTGGTGGG	-3′
	Reverse Primer	5′-	TGCTTTAGCTCGGTTGTG	-3′
ME1	Forward Primer	5′-	GTTGCCCTTGGTGTTGT	-3′
	Reverse Primer	5′-	GGATAAATGGTGGCTGTC	-3′
Nanog	Forward Primer	5′-	TCCAGCAGATGCAAGAACTCTCCA	-3′
	Reverse Primer	5′-	CACACCATTGCTATTCTTCGGCCA	-3′
SOX2	Forward Primer	5′-	ATGACCAGCTCGCAGACCTAC	-3′
	Reverse Primer	5′-	TTGACCACCGAACCCATGGAG	-3′
